# The Utility of Salvage Radiotherapy for an Inoperable Inguinal Recurrence of Squamous Cell Carcinoma of the Penis

**DOI:** 10.7759/cureus.48815

**Published:** 2023-11-14

**Authors:** W. C. Ian Janes, Paul Johnston, Michael Organ, John Thoms, Eduardo Gaviolli

**Affiliations:** 1 Medicine, Memorial University of Newfoundland, St. John's, CAN; 2 Urology, Memorial University of Newfoundland, St. John's, CAN; 3 Radiation Oncology, Memorial University of Newfoundland, St. John's, CAN

**Keywords:** lymph nodes, inguinal, metastases, penile cancer, salvage radiotherapy

## Abstract

Penile cancer is a rare genitourinary malignancy for which limited treatment options exist beyond primary surgical resection. Metastatic lymphadenopathy represents a particularly poor prognosis with a lack of literature to suggest the effectiveness of radiation or systemic therapies. Our case documents an inguinal recurrence of penile squamous cell carcinoma not amenable to surgical intervention demonstrating complete response to salvage radiotherapy in the palliative setting. These observations propose the need for further research around the utility of radiotherapy in the management of metastatic penile malignancies.

## Introduction

Carcinoma of the penis is a rare and debilitating malignancy for which there is limited evidence to support treatment modalities aside from primary surgical resection [1,2]. Regional lymphatic spread is often the most powerful predictor of prognosis and further limits potential treatment options in the context of existing comorbidities [3]. Radiation therapy, despite viability in the treatment of various genitourinary malignancies, is rarely employed in the management of penile cancer beyond early-stage presentations or neoadjuvant control of nodal disease [1,3,4]. Salvage radiotherapy has not previously been considered in the metastatic setting for penile cancer [5]. Herein, we present a medically complex case of penile cancer with inoperable inguinal metastases treated with salvage radiation, displaying complete response in the palliative setting.

## Case presentation

A 46-year-old male with a known history of ulcerative colitis presented in January 2020 with complaints of a painless lesion on the glans penis present for approximately two months' duration. A punch biopsy of the area was obtained indicating invasive squamous cell carcinoma (SCC) and warranting urgent urologic referral for surgical consideration. During the course of the surgical work-up, the patient was found to have significantly elevated liver enzymes which led to the additional diagnosis of alcohol-related cirrhosis with associated elements of non-alcoholic steatohepatitis. Definitive surgical intervention was undertaken via partial penectomy in early February 2020 with pathology reported as human papillomavirus (HPV)-related, well-differentiated SCC consistent with pT2NX disease with negative margins and without lymphovascular or perineural invasion. Considering the favourable pathology without clinical evidence of pelvic adenopathy, extant guidelines deemed the patient appropriate for active surveillance with routine physical examination and computed tomography (CT) imaging.

**Figure 1:**
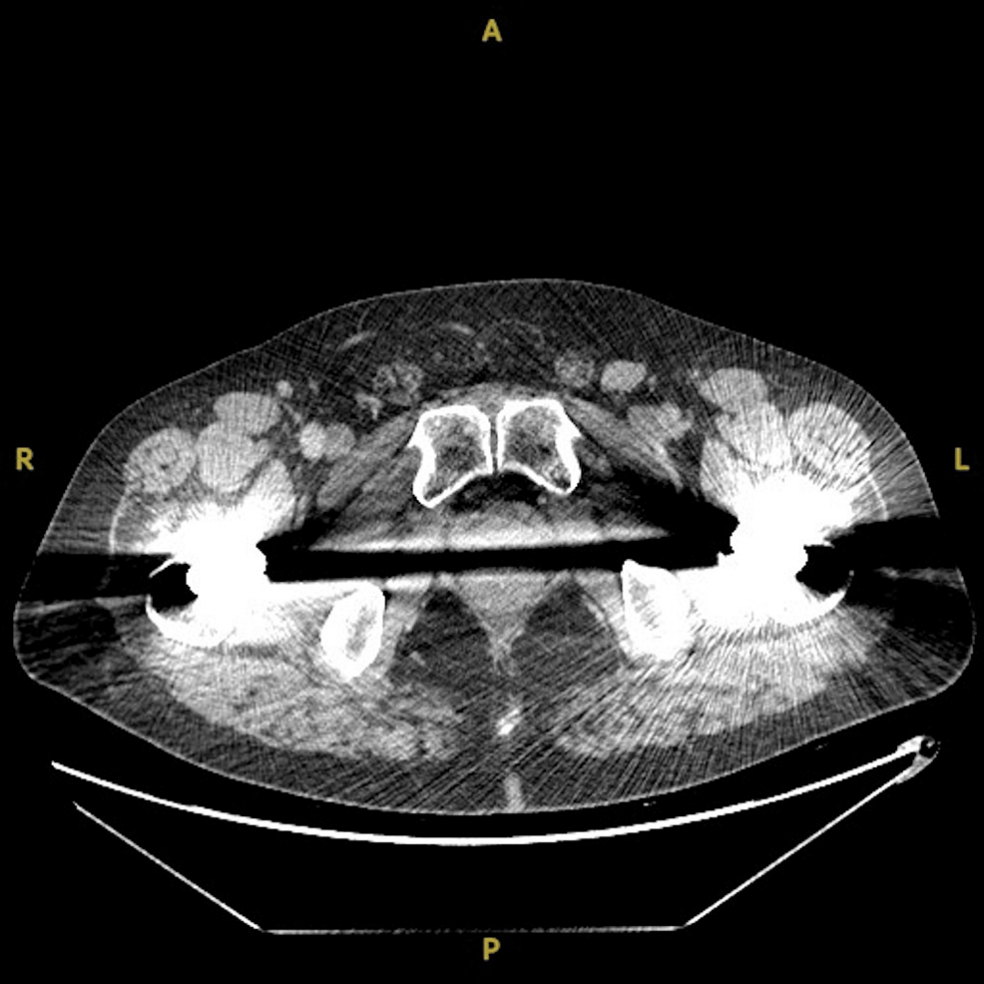
Pre-salvage radiotherapy CT chest/abdomen/pelvis demonstrating inguinal lymphadenopathy. CT: computed tomography

After approximately one year on surveillance protocol, multiple enlarging inguinal lymph nodes were noted on follow-up imaging, the largest measuring 2 cm in size with the confirmation of metastatic disease via ultrasound-guided biopsy (Figure 1). At this point, the patient had developed advanced liver disease with various associated complications over the preceding year, including variceal bleeding, abdominal ascites requiring frequent paracenteses, and portal hypertension. These comorbidities paired with the significant association of bilateral inguinal lymphadenectomy with lymphedema of the lower extremities precluded the patient from further surgical candidacy. Review by medical oncology deemed the patient a poor candidate for systemic therapy due to complex comorbid interactions and elevated risk for potential life-threatening complications.

**Figure 2:**
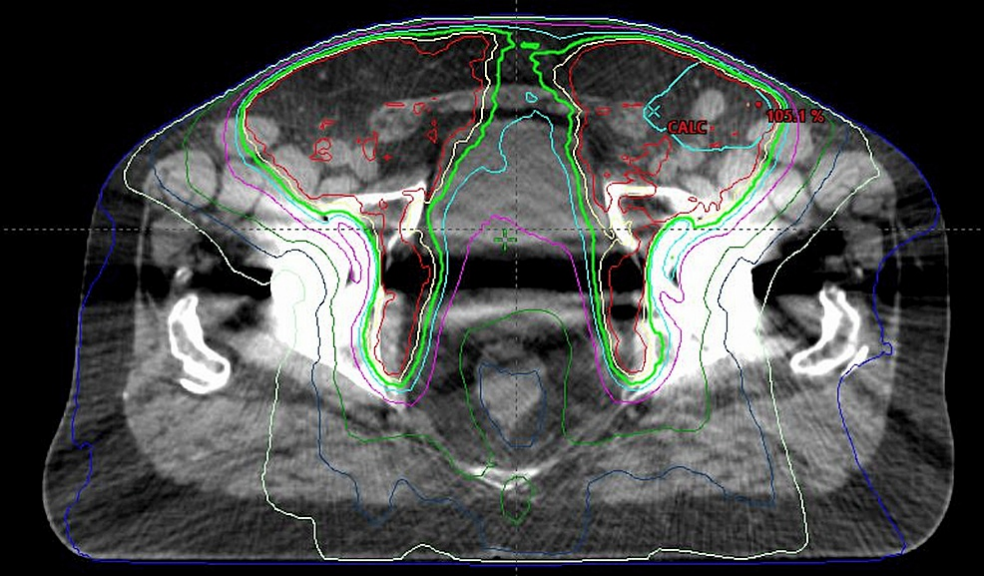
Utilized radiation treatment plan for the inguinal recurrence of penile SCC. SCC: squamous cell carcinoma

Given the patient's known history of inflammatory bowel disease (IBD) and associated risks for radiation toxicity, there were significant consideration and interdepartmental discussion surrounding the utility of radiotherapy in the present case. Ultimately, the patient elected to undergo salvage treatment with three-phase palliative radiotherapy. Treatment was delivered as 3,600 cGy in 18 fractions to the pelvic lymph nodes extending to the bifurcation of the common iliac artery with a further 4,600 cGy over 23 fractions administered to the inguinal nodes with boost to 5,400 cGy in 27 fractions for involved lymph nodes (Figure 2).

**Figure 3:**
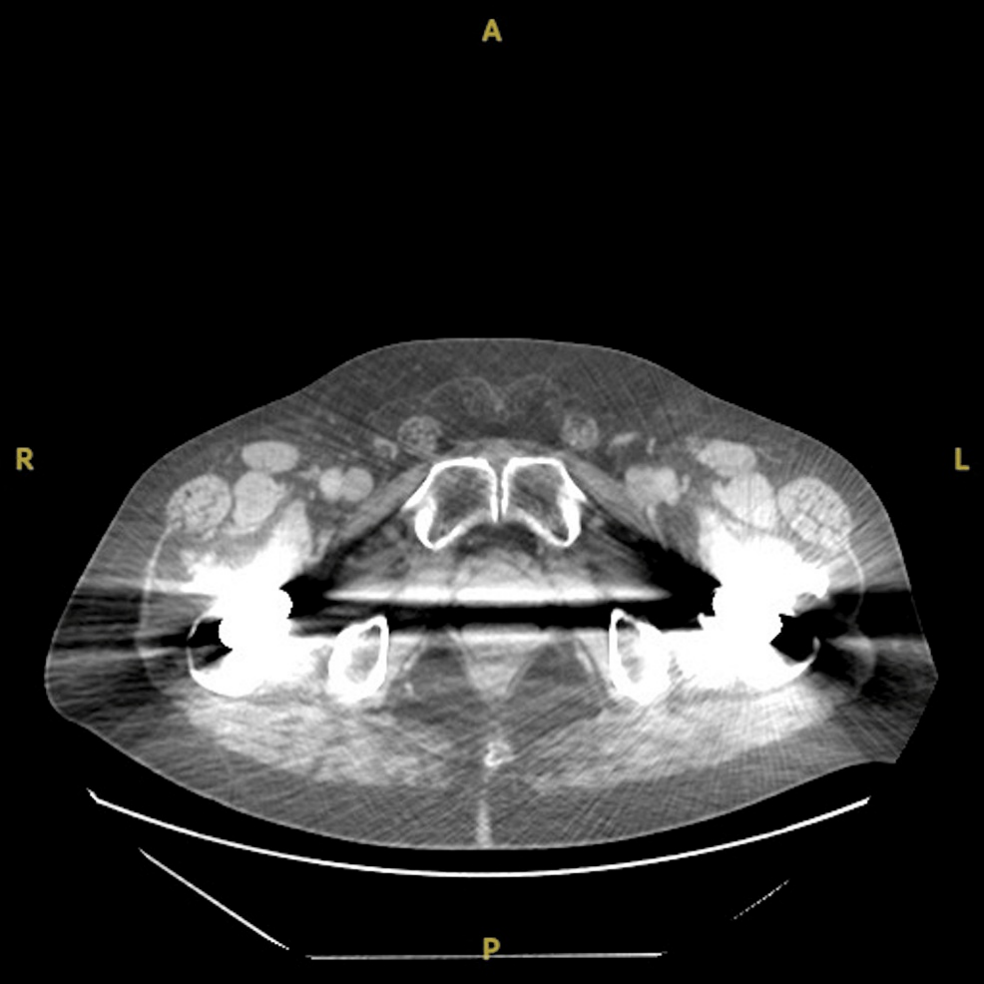
Post-salvage radiotherapy CT chest/abdomen/pelvis showing the complete eradication of inguinal disease. CT: computed tomography

The patient completed treatment in June 2021 with the complete eradication of nodal disease on CT scan without evidence of new lesions. Follow-up imaging over the preceding 15 months continued to show complete response to radiation treatments and absence of treatment-related side effects or toxicity (Figure 3). Unfortunately, the patient was deceased as of November 2022 secondary to complications of his liver disease; however, continued complete response to radiotherapy was seen at the time of death.

## Discussion

Penile SCC remains a rare entity with continuously evolving treatment guidelines grounded in the outcomes of small heterogeneous studies and retrospective reviews. Currently, the functional treatment paradigm for these malignancies specifies limited role for therapies beyond primary surgical resection and inguinal lymphadenectomy [2,6]. Locally advanced and metastatic diseases are associated with dismal outcomes, and inguinal recurrence carries a particularly poor prognosis, with decreasing overall survival dependent on the burden of nodal disease [7,8].

Traditionally, radiotherapy has been employed infrequently in the radical management of early-stage, localized penile cancer or in the provision of (neo)adjuvant control of nodal disease [2,3]. These modalities have further been considered to provide palliative benefit ﻿in patients with unresectable inguinal metastases, often in conjunction with systemic therapies [9]. In cases of metastatic penile cancer, significant debate remains around the utility of adjuvant radiotherapy in the prevention of post-lymphadenectomy nodal disease, with varying results across multiple studies [2,10-12]. Franks et al. reported that adjuvant radiotherapy improved the overall survival in patients with penile cancer following lymphadenectomy compared with those receiving high-grade palliative radiation [12]. Conversely, a more recent systematic review found insufficient evidence to support the use of adjuvant inguinal radiotherapy, citing inconsistent recurrence rates ranging from 10% to 92% [11]. Both of these prior works cited high risk of bias paired with low quality of evidence secondary to retrospective design and small sample sizes.

As such, there is a further lack of literature pertaining to the utility of radiation therapy for confirmed nodal disease from penile primaries with current guidelines discouraging use in these settings [3,11]. Salvage radiotherapy has been frequently utilized in the treatment of biochemically recurrent prostate cancer; however, the same concepts have rarely been explored for nodal spread in those with penile cancer. Zong et al. reported a case of penile cancer with unresectable malignant lymphadenopathy demonstrating significant response following pelvic irradiation [13]. Similarly, our case documents an inoperable inguinal recurrence of penile cancer which showed unexpected complete response to salvage radiotherapy in what was deemed a palliative setting. Of note, our patient had less aggressive disease without the further extensive metastatic spread reported by the prior authors which may have contributed to our overall favourable result. Additionally, the employed palliative radiotherapy regimen in our case was extrapolated from that utilized as standard of care in the management of node-positive vulvar cancer. Prior research has documented improved survival for vulvar SCC with pelvic metastases in those receiving adjuvant radiotherapy to the bilateral groins when compared with primary lymphadenectomy alone [4]. Given the similarity of these sex-specific malignancies paired with the observed response in our case, it is possible that radiation therapy may have further utility in the management of locally advanced penile cancer.

There was significant debate surrounding the ability to safely utilize radiotherapy in our patient due to his history of IBD as initial literature on this topic indicated likelihood of severe toxicities nearing 50% [14]. However, more recent reviews have indicated that IBD should not be considered contraindicatory as the evolution of radiotherapy techniques has decreased the rate of reported toxicities [15,16]. Paired with the extenuating circumstances of our patient's disease and the lack of surgical or systemic treatment options, radiotherapy was deemed an acceptable undertaking. It is important to consider that the presence of HPV-related disease in our patient may have affected response to radiotherapy as previous research has indicated positive prognostic and predictive implications of HPV status in females with vulvar malignancies [17]. Additionally, the extent of disease spread was confined to the inguinal lymph nodes which may have been an additional predictor of our patient's response to radiotherapy. It is difficult to interpret long-term outcomes due to the patient's untimely and unrelated death.

## Conclusions

The management of penile cancer is continuously evolving. We have presented a case of penile cancer with inoperable inguinal recurrence for which salvage radiotherapy resulted in curative effect and may provide important consideration in future medically complex cases.
